# Advances in Nanotoxicology: Health and Safety

**DOI:** 10.3390/nano16171096

**Published:** 2026-09-01

**Authors:** Natalia Krasteva, Milena Georgieva

**Affiliations:** 1 Institute of Biophysics and Biomedical Engineering, Bulgarian Academy of Sciences, 1113 Sofia, Bulgaria; 2 Laboratory of Molecular Genetics, Epigenetics and Longevity, Institute of Molecular Biology “Roumen Tsanev, Bulgarian Academy of Sciences, 1113 Sofia, Bulgaria

## 1. Introduction

The rapid development of nanotechnology has resulted in the widespread incorporation of engineered nanomaterials into biomedical, industrial, and consumer applications, including drug delivery systems, imaging agents, therapeutic platforms, cosmetics, food packaging, and biosensing technologies [1,2,3,4,5,6,7,8,9,10]. Therefore, the better the distinctive physicochemical properties of nanomaterials are the greater their opportunities to address major technological and medical challenges. However, the increasing use of those materials also raises important questions regarding their potential effects on human health and the environment [11,12,13,14,15]. Consequently, understanding how engineered nanomaterials interact with biological systems and how these interactions translate into beneficial or adverse biological effects has become a central objective of modern nanotoxicology, which has progressively moved beyond the conventional assessment of whether a nanomaterial is “toxic” or “nontoxic” toward a more mechanistic and context-dependent understanding of nanoparticle–biological interactions [16]. This shift reflects the recognition that a single material characteristic or exposure parameter rarely determines biological responses. Rather, they emerge from the complex interplay between nanomaterial physicochemical properties, their transformations within biological environments, exposure conditions, and the intrinsic characteristics of cells, tissues, and organisms. Such a framework is essential for distinguishing intrinsic material-associated hazards from biological responses arising from changes in particle state, degradation, dissolution, aggregation, or interactions with biomolecules and biological barriers. A particularly important aspect of this complexity is the dynamic nature of the nanobio interface [17]. Once introduced into a biological environment, nanoparticles may undergo substantial changes in their physicochemical properties, including aggregation or agglomeration, surface modification, dissolution, protein corona formation, cellular uptake, intracellular trafficking, and transformation [18]. These processes can alter particle bioavailability, biodistribution, cellular interactions, and ultimately biological activity. As a result, the biological behavior of a nanomaterial in vivo may differ considerably from that predicted based on its pristine properties alone.

Nanoparticle-induced biological responses are further influenced by a broad range of interconnected factors, including particle size and morphology, surface charge and functionalization, chemical composition, concentration and dose metrics, exposure duration, aggregation state, dissolution, route of exposure, biodistribution, and the presence of biological or pathological conditions [19,20,21,22,23]. The biological model itself is also a critical determinant of outcome, as differences in cellular phenotype, tissue organization, metabolic activity, barrier properties, and disease status can substantially modify nanoparticle responses. Accordingly, nanoparticle toxicity should not be considered an intrinsic and universal property of a material class, but rather as an outcome resulting from the interaction between material characteristics, exposure conditions, and biological context [24,25]. This complexity also presents important methodological challenges for the field. Differences in nanoparticle synthesis and physicochemical characterization, dispersion and exposure protocols, dose metrics, experimental conditions, biological models, and selected toxicity endpoints can contribute to substantial variability between studies and complicate the comparison and reproducibility of findings.

In particular, conventional mass-based exposure metrics may not fully capture the biological relevance of nanoscale materials, for which particle number, surface area, dissolution, or delivered cellular dose may provide complementary information. Robust nanotoxicological assessment therefore requires careful control and characterization of the material throughout the experimental process, together with the integration of complementary physicochemical, cellular, molecular, and organism-level endpoints [26]. These considerations are increasingly important not only for hazard identification but also for the development of safer and more effective nanotechnologies. The concept of safe-by-design nanotechnology emphasizes identifying and optimizing potentially hazardous material characteristics at an early stage of development, rather than assessing safety only after a material has been produced [27]. Achieving this goal requires a deeper understanding of the relationships between specific physicochemical characteristics and biological responses, supported by mechanistic toxicology, appropriate exposure assessment, and predictive approaches. Such knowledge can help distinguish properties that drive adverse effects from those responsible for desirable biological functions and can thereby support the rational design, evaluation, and translation of nanomaterials for biomedical and other applications.

Against this scientific background, the Special Issue “Advances in Nanotoxicology: Health and Safety” was conceived to bring together recent research addressing key aspects of nanomaterial safety and nanoparticle biological interactions. The studies presented in this issue encompass a diverse range of nanomaterial platforms, including silicon quantum dots, superparamagnetic iron oxide nanoparticles, silver nanoparticles, metal oxide nanoparticles, and graphene oxide-based nanomaterials. Through complementary experimental approaches, these studies investigate different aspects of nanomaterial behavior and biological response, including cellular and molecular mechanisms of toxicity, oxidative and inflammatory effects, genotoxicity, interactions with biological systems, and factors influencing nanoparticle safety and efficacy. The diversity of materials, biological models, and analytical approaches represented in the issue reflects the multidisciplinary nature of contemporary nanotoxicology. It illustrates the need to integrate advanced material characterization with mechanistic biological assessment. By bringing these complementary perspectives together, this Special Issue aims to contribute to a more comprehensive and predictive understanding of nanomaterial-associated risks and to support the continued development of nanotechnology in a manner that balances technological innovation with human health and safety.

## 2. Overview of Published Articles

Cristian et al. (Contribution 1) investigated the acute in vivo hepatic and renal toxicity of laser-ablated silicon quantum dots (SiQDs) in mice, demonstrating dose- and time-dependent oxidative stress, disruption of antioxidant defenses, histopathological alterations, and inflammatory responses, particularly at doses above 10 mg/kg. Importantly, doses below 10 mg/kg did not induce significant hepatic or renal toxicity or genotoxicity, providing valuable information for defining safety margins and highlighting the importance of dose- and organ-specific assessment in the development of silicon-based nanomaterials for biomedical applications.

Haase et al. (Contribution 2) investigated the safety of repeated intravenous administration of very small superparamagnetic iron oxide particles (VSOP-T) at 100 μmol Fe/kg as magnetic resonance angiography and molecular imaging agents in atherosclerotic mice. Although VSOP-T accumulated in the liver and spleen, repeated exposure at an imaging-relevant dose did not cause significant organ pathology, alterations in iron homeostasis or serum lipids, or aggravation of atherosclerotic plaque progression. These findings highlight the importance of evaluating nanomaterial safety under repeated-exposure conditions and in disease-relevant models, supporting the further development of VSOP-T for longitudinal cardiovascular imaging.

Pitchai et al. (Contribution 3) demonstrated that metabolic syndrome significantly increases susceptibility to pulmonary inflammation following exposure to 20 nm silver nanoparticles (AgNPs; 50 μg), emphasizing the importance of host health status in determining nanotoxicological outcomes. Treatment with specialized pro-resolving mediators—Resolvin E1, Protectin D1, and Maresin 1 (400 ng)—differentially reduced AgNP-induced neutrophilic inflammation and pro-inflammatory signaling, highlighting inflammation-resolution pathways as potential therapeutic targets and the need to consider susceptible populations in nanoparticle safety assessment.

Meindl et al. (Contribution 4) investigated the potential of metal oxide nanoparticles, including silica, titanium dioxide, and zinc oxide nanoparticles, to influence skin sensitization responses. Through a combination of advanced in vitro models involving keratinocytes and immune cell systems, the authors demonstrated that nanoparticles may modify sensitization responses depending on nanoparticle type and cellular context. Their findings underline the importance of developing physiologically relevant alternative testing approaches that better reproduce complex biological interactions and improve the predictive capacity of nanotoxicological assessments.

The contribution of Keremidarska-Markova et al. (Contribution 5) expands the understanding of graphene oxide (GO)-based nanomaterial safety, addressing an important class of nanomaterials increasingly explored for biomedical applications, including photothermal therapy and drug delivery. The study provided a comprehensive evaluation of the effects of GO and polyethylene glycol-modified GO (GO-PEG) using complementary models at organ, cellular, and subcellular levels. The authors demonstrated that both nanomaterials influenced cardiac contractility, increased reactive oxygen species production in skeletal muscle cells, and modulated hepatic enzyme activity, with some effects altered following near-infrared irradiation. Additionally, mitochondrial analyses revealed concentration-dependent effects on ATPase activity. This multifaceted approach highlights the importance of evaluating nanomaterial biological effects across multiple levels of organization, particularly when considering multifunctional nanoplatforms designed for biomedical applications.

## 3. Conclusions

The contributions to this Special Issue collectively reinforce the view that nanotoxicological outcomes arise from complex interactions between nanomaterial properties, exposure conditions, and biological context. Rather than representing an intrinsic and uniform characteristic of a given nanomaterial class, toxicity is shaped by factors such as composition, surface characteristics and functionalization, physicochemical behavior in biological environments, dose and duration of exposure, and the properties of the biological system under investigation. This complexity highlights the importance of combining rigorous physicochemical characterization with complementary biological and mechanistic approaches to obtain a meaningful assessment of nanomaterial safety.

An important implication is the need to consider biological context when evaluating potential risks. Cellular phenotype, tissue and organ characteristics, disease status, and individual susceptibility can substantially influence nanoparticle fate and biological effects. Accordingly, the use of advanced in vitro systems, relevant in vivo models, and mechanistic molecular analyses can provide complementary information on processes including oxidative stress, inflammation, immune modulation, mitochondrial dysfunction, genotoxicity, and tissue-specific responses. Such integrated approaches can improve the interpretation of experimental findings and strengthen their relevance to potential human exposure and biomedical applications.

The ultimate goal of nanotoxicology extends beyond hazard identification toward enabling the development of nanomaterials that retain their intended therapeutic or technological functions while minimizing adverse effects. A more detailed understanding of the relationships between physicochemical characteristics, biological transformations, and mechanisms of toxicity can support rational risk assessment and contribute to safe-by-design strategies. In this context, mechanistic evidence is particularly valuable for identifying material characteristics that can be modified to improve safety without compromising functionality.

Despite substantial progress, several challenges remain. These include predicting long-term and chronic effects, assessing repeated or low-level exposures, harmonizing and standardizing testing protocols, selecting biologically relevant dose metrics, and establishing robust and reproducible relationships between physicochemical properties and biological outcomes. Addressing these challenges will require continued integration of nanomaterial science with molecular and cellular toxicology, advanced experimental models, computational and predictive approaches, and clinically relevant systems. Such multidisciplinary efforts will be essential for advancing nanotoxicology from descriptive hazard assessment toward a more predictive framework capable of supporting the safe and responsible development of next-generation nanotechnologies.
